# Human disturbance, preys and refuge cover shape top predator movements in anthropogenic landscapes

**DOI:** 10.1093/beheco/arag037

**Published:** 2026-04-13

**Authors:** Iago Ferreiro-Arias, Iris Martínez, Emilio José García, Vicente Palacios, Víctor Sazatornil, Alejandro Rodríguez, José Vicente López-Bao, Luis Llaneza

**Affiliations:** Department of Conservation Biology and Global Change, Estación Biológica de Doñana, CSIC, Américo Vespucio 26, Sevilla 41092, Spain; Department of Biogeography and Global Change, Museo Nacional de Ciencias Naturales, CSIC, C. de José Gutiérrez Abascal 2, Madrid 28006, Spain; A.RE.NA. Asesores En Recursos Naturales, S.L. Perpetuo Socorro n°12-Entresuelo, 2B, Lugo 27003, Spain; ARCA People and Nature, S.L. Tenderina 69, 1A, Oviedo 33010, Spain; ARCA People and Nature, S.L. Tenderina 69, 1A, Oviedo 33010, Spain; Conservation Biology Group, Landscape Dynamics and Biodiversity Programme, Forest Science and Technology Centre of Catalonia (CTFC), Sant Llorenç de Morunys, km 2, Solsona 25280, Spain; Department of Conservation Biology and Global Change, Estación Biológica de Doñana, CSIC, Américo Vespucio 26, Sevilla 41092, Spain; Biodiversity Research Institute (University of Oviedo – CSIC – Principado de Asturias), Oviedo University, c/ Gonzalo Gutiérrez Quirós s/n, Mieres 33600, Spain; A.RE.NA. Asesores En Recursos Naturales, S.L. Perpetuo Socorro n°12-Entresuelo, 2B, Lugo 27003, Spain; Grupo de Investigación en Biología Evolutiva (GIBE), Facultade de Ciencias, Área de Zooloxía, Universidade da Coruña, Campus da Zapateira s/n, A Coruña 15071, Spain

**Keywords:** *Canis lupus*, daily distance, domestic ungulates, human disturbance, net displacement, straightness index, vegetation cover

## Abstract

Studies on wolf movement have focused on the effects of different isolated drivers, such as intrinsic traits or prey abundance, in wild settings. Consequently, a holistic understanding of how several factors jointly shape daily movement patterns, particularly in heavily anthropized landscapes, remains lacking. To examine the joint effect of intrinsic factors, food availability, human disturbance and refuge cover, we equipped 26 individuals with GPS-GSM collars in NW Iberia, one of the most anthropized areas of their range. We obtained 54,721 locations and 4,560 daily trajectories from positions taken every 2 h. We calculated 3 daily movement metrics: daily distance, net displacement, and straightness index. Using Bayesian regression models and variance partitioning, we assessed the relative contribution of intrinsic traits and external factors related to food availability, anthropogenic disturbance and refuge cover on wolf movement. Wolves traveled a mean daily distance of 9 km, with a mean net displacement of 3.8 km. Anthropogenic disturbance, particularly the interaction between human population and settlement density, was the main driver of movement reduction. Paved road density had a negative effect on movement, but this was mitigated by the extent and cohesion of refuge cover. Wolves feeding on livestock traveled shorter daily distances and net displacements compared with those feeding on wild ungulates. Our findings highlight the importance of the interplay between trophic resources, anthropogenic disturbance, and refuge availability in shaping daily wolf movements. In human-dominated landscapes, wolves shorten their movements to minimize exposure and mortality risk where close food and refuge are available.

## Teaser

Wolves reduce their daily distances and net displacements in landscapes dominated by humans and their infrastructures, averaging about 9 and 4 km, respectively. Vegetation cover helps to buffer these effects, allowing longer movements despite disturbance. Wolf movements vary with prey type, where individuals feeding on livestock cover shorter distances than those feeding on wild prey. Wolves likely adapt their behaviors by balancing mortality risk by humans, food availability, and vegetation cover in human-dominated environments.

## Introduction

Understanding wildlife movement is a central topic in behavioral ecology, given its significance for ecological processes at the individual, population, and ecosystem levels (eg, foraging, dispersal, migration) (Blanco and Cortés 2007; Bauer and Hoye 2014; Ylitalo et al. 2021). Movement patterns emerge from the interplay between intrinsic factors, such as physiological state or life-history stage, and extrinsic factors including resource distribution, landscape structure, or climatic conditions (Nathan et al. 2008). In the Anthropocene, a critical challenge is to understand how this interplay is reconfigured by novel, human-induced extrinsic pressures, which can simultaneously provide attractive subsidies and impose pervasive risks (Gomez et al. 2025). In landscapes heavily modified by humans, the displacements of wildlife expressed as the straight-line distance between the start and end points of a trajectory are halved compared with displacements in more natural areas, indicating a significant constraint on animal movement (Tucker et al. 2018). While often attributed to habitat modification, movement reductions are generally driven by human activity, as evidenced by a 73% global increase in mammal displacements during COVID-19 lockdowns (Tucker et al. 2023). Importantly, these shifts in movement patterns can have profound implications by altering resource availability, reproductive opportunities, and survival rates, while also influencing overall ecosystem functioning (Fahrig 2007; Doherty et al. 2021; Tucker et al. 2021).

Changes in movement patterns have been extensively explored for many large carnivores (Evans et al. 2019; Ellington et al. 2020; Suraci et al. 2020; Habib et al. 2021). The movement strategies of major carnivore families are significantly shaped by their distinct evolutionary histories, which establish deep-rooted behavioral legacies for territory use and foraging movements (Fagan et al. 2025). However, within these phylogenetic constraints, movement patterns are also highly flexible, modulated by life-history stages (Barry et al. 2020; O’Neill et al. 2020; Bista et al. 2021; Thorsen et al. 2022) and resource availability (Frame et al. 2004; Petroelje et al. 2019; Barceló et al. 2025). In human-dominated landscapes, carnivore movement may be further influenced by a tradeoff between exploiting resources and avoiding anthropogenic threats (Lamb et al. 2020; Barceló et al. 2025). Human activity can reshape carnivore movement by providing attractive resources, such as food subsidies that could reduce displacements and repulsive forces like persecution or infrastructures that induce avoidance and might truncate movement paths (Petroelje et al. 2019; Lamb et al. 2020; Barceló et al. 2025). As a consequence, the expected result is a reconfiguration of the movement patterns of carnivores in human-dominated landscapes, which are therefore not a simple reflection, but a complex expression of species-specific ecology and evolutionary history and the dynamic interaction with the human-shaped environment (Gomez et al. 2025). Hence, carnivores in anthropogenic landscapes constitute an informative model system to dissect the complex interplay between intrinsic behavioral factors and extrinsic human pressures that collectively determine movement patterns.

Within Carnivora, a compelling model for studying these dynamics is the gray wolf (*Canis lupus*), a holartic generalist top-predator whose persistence from pristine to heavily anthropized landscapes provide us with key insights about how intrinsic traits and extrinsic pressures shape carnivore movement (Ciucci et al. 1997; Jedrzejewski et al. 2001; Eggermann et al. 2009; Mech and Cluff 2011; Rio-Maior et al. 2018; Petroelje et al. 2019; Kirilyuk et al. 2021; Dickie et al. 2022). Intrinsic factors such as social status, sex, age, and reproductive season cause significant variation in traveled distances by wolves. For instance, dispersing individuals and adults typically cover longer distances, while breeding wolves exhibit restricted movements during the denning season (Rio-Maior et al. 2018; Petroelje et al. 2019; Ellington et al. 2020). Extrinsically, food availability is a primary driver of carnivore movement, and wolves strategically adapt their movement patterns to exploit different resource distributions (Frame et al. 2004; Petroelje et al. 2019; Ylitalo et al. 2021). In natural systems, the scattered and unpredictable nature of wild prey occurrence necessitates a “search-and-pursue” strategy, leading to longer travel distances (Jedrzejewski et al. 2001). Conversely, the spatial aggregation of livestock provides a predictable, clumped anthropogenic subsidy, enabling a shift to a more efficient “patrol-and-exploit” strategy that significantly reduces movement rates (Ciucci et al. 1997; Kusak, Skrbinšek and Huber 2005; Petroelje et al. 2019).

The exploitation of anthropogenic trophic resources exposes carnivores to a suite of mortality risks, since navigating these landscapes increases the likelihood of human-induced mortality, both from deliberate persecution in retaliation for livestock predation or incidental causes such as vehicle collisions on road networks (Chavez and Gese 2006 ; Lamb et al. 2020; Whittington et al. 2022; Morales-González et al. 2025). Particularly, the response of wolves to these risks are complex and scale-dependent, exhibiting substantial inter- and intra-population variability (Zimmermann et al. 2014; Milleret et al. 2019; Barry et al. 2020; Ferreiro-Arias et al. 2024). Notably, these spatio-temporal responses of wolves are an example of key context-dependent avoidance strategies that can occur at very fine scales in carnivores, promoting a dynamic coexistence in shared landscapes (López-Bao et al. 2016; Ferreiro-Arias et al. 2021). Gray wolves in particular demonstrate a remarkable capacity to finely tune their behavior, dynamically partitioning space and time with humans (Ferreiro-Arias et al. 2024). For instance, wolves can select linear infrastructures with low human activity to facilitate their movements and travel longer distances (Dickie et al. 2017; DeMars and Boutin 2018). Conversely, even if paved roads with high traffic volume often induce avoidance and reduce landscape permeability (Whittington et al. 2004; Rio-Maior et al. 2018), wolves are able to select times when traffic volume is reduced to cross those barriers (Dennehy et al. 2021).

Beyond these factors, the use of vegetation cover also emerges as a crucial factor buffering risk exposure of carnivores in human-dominated landscapes, mitigating the adverse effects of both human activity and infrastructure occurrence (Llaneza et al. 2016; Sazatornil et al. 2016; Grilo et al. 2019; Cimatti et al. 2021; Ferreiro-Arias et al. 2024; Jansson et al. 2024). In human-dominated landscapes, vegetation patches vary widely in their degree of human alteration, ranging from croplands and forest plantations to remnants of natural vegetation and protected areas embedded in a highly transformed matrix (Ferreira et al. 2018). While the functionality of modified habitat patches may be limited for some specialist carnivores (Ferreira et al. 2018; Bista et al. 2021), wolves often demonstrate remarkable adaptability, utilizing such patches effectively even in highly transformed landscapes (Massolo and Meriggi 1998; Houle et al. 2010; Llaneza et al. 2018; Grilo et al. 2019; Cimatti et al. 2021; Kudrenko et al. 2023). However, it is still unclear how different attributes of refuge cover such as patch size, density or cohesion influence different components of wolf movement and buffer the potential negative effects of human infrastructure.

To date, research on wolf movement has typically focused on isolating the effects of single drivers, be they intrinsic (eg, social status) or extrinsic (eg, prey distribution, human infrastructure) (Jedrzejewski et al. 2001; Kusak et al. 2005; Rio-Maior et al. 2018; Kirilyuk et al. 2021; Whittington et al. 2022). While informative, these approaches cannot quantify the interaction between factors or reveal the primary determinants of daily movement patterns. To move from a compartmentalized to a holistic understanding, a comprehensive framework that evaluates the relative importance and joint effects of key drivers is essential. Here, we explore how individual characteristics such as age, sex, or social status, as well as extrinsic factors related to anthropogenic disturbance, refuge cover and prey availability modulate different aspects of wolf daily movements in one of the most anthropized regions of their distribution range, the Iberian Peninsula (Llaneza et al. 2012). Specifically, we aim: (1) to provide a description of daily movement patterns and distances traveled by wolves; (2) to quantify the relative importance of intrinsic and extrinsic factors on different attributes of wolf daily movements; and (3) to understand the nature of the relationship of different movement attributes with predictors related to anthropogenic disturbance, landscape attributes, refuge cover and prey availability.

## Material and methods

### Study area

**Figure 1:**
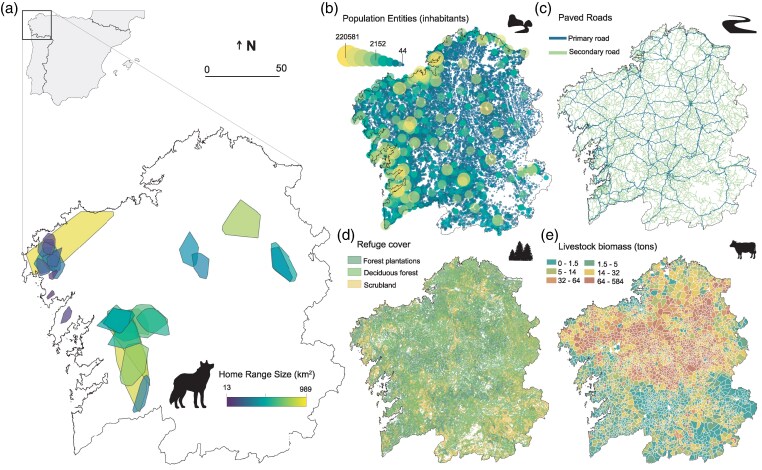
Study area in north-western Spain showing: a) the location of Galicia within the Iberian Peninsula and the distribution of home ranges for the studied individuals, color-coded according to the size of their respective home ranges; b) the distribution of human population entities, with circle size and color representing the number of inhabitants; c) the distribution of primary and secondary roads; d) the distribution of potential vegetation patches functionally serving as refuges for wolves; and e) the distribution of livestock biomass.

This study was conducted in Galicia, north-western Spain (∼30,000 km^2^), where wolves currently occupy most of its extension (ca. 90%) and have shown a stable trend in the estimated number of breeding packs over the past decade (Llaneza et al. 2015, 2022) (Fig. 1a). Galicia is characterized by a high human population density (ca. 94 inhabitants/km^2^) widely dispersed across the region, with an average of 3 settlements per km^2^ and a road density of 3.5 km/km^2^ (INE 2010) (Fig. 1b, c). The Galician landscape is dominated by agricultural land uses and extensive forest plantations (Calvo-Iglesias et al. 2009; Llaneza et al. 2012; Diaz-Maroto and Diaz-Maroto 2018). The region is primarily characterized by stands of *Pinus spp*. and *Eucalyptus spp*. plantations and pastures for livestock, collectively covering about 55% of the area. Scrublands and deciduous forests make up ca. 17% and 10% of the landscape, respectively (SIOSE National Technical Team 2022) (Fig. 1d). The spatial distribution of wild and domestic ungulates translates into marked regional differences in the availability of different prey types. Regarding wild ungulates, roe deer (*Capreolus capreolus*) and wild boar (*Sus scrofa*) are abundant in the eastern half of the region where they are the primary prey of wolves, while in western sectors wild ungulates occur at lower densities or they are absent (López-Bao et al. 2013). On the other hand, an ancient breed of free-ranging horses (*Equus caballus*) occurs in Galician heathlands following the legacy of traditional husbandry practices, and serves as the main prey for wolf packs in specific regions of the west and north (López-Bao et al. 2013 ; Lagos and Bárcena 2018). Finally, wolves prey upon domestic ungulates mainly in the north-western and western sectors (Lázaro-Fuentes 2014). Galicia has a vast development of semi-extensive livestock facilities sustaining an average of 4.7 livestock farms per km^2^ (INE 2009), which results in an broad distribution of domestic ungulates where cattle (*Bos taurus*) and sheep and goat (*Ovies aries* and *Capra hircus*) reach average densities of 37.9 (range: 0 to 685.2) and 13.5 heads per km^2^ (range: 0 to 1,124.6), respectively (Fig. 1e) (INE 2009).

### Wolf monitoring and data collection

Data from this study comes from a sample of 26 wolves captured and monitored with GPS-GSM collars (Tellus T3H and T5H models from Followit, Sweden) between 2006 and 2014 (Additional File 2, Table S1). Monitored individuals represent a random sample of the wolf population in the study area as most wolves were solitary individuals or belonged to 15 different packs distributed throughout the region (Fig. 1), with a balanced representation of sexes and age classes (Additional File 2, Table S1). Wolves in this study were captured using Belisle leg-hold snares (Edouard Belisle, Saint Veronique, PQ, Canada) and immobilized via intramuscular injection of medetomidine (0.10 mg/kg; Dormitor, Merial, Lyon, France), with reversal achieved through atipamezole (Revertor, Merial, Lyon, France). All capture and handling procedures complied with animal welfare regulations (permits 19/2006, 71/2009, 86/2011, and 95/2013 from the Regional Government of Galicia, and Spanish Decree 53/2013). Captured wolves were clinically healthy at the time of capture, presenting only minor trapping-related lesions such as skin abrasions. GPS-GSM collars were programed to record wolf locations with varying schedules (ie, every 20-min, 1-h and 2-h), so we used locations recorded at 2-h intervals to standardize the data and ensure comparability across individuals. GPS-GSM devices recorded 54,721 wolf locations, covering approximately 109,442 h of sampling and 4,560 raw daily trajectories. Mean (± SD) of sampling days per individual was 193 (±100) days (range: 52 to 397) (Additional File 2, Table S1).

For each GPS position, we extracted information of date, time and coordinates in decimal degrees (WGS84). Sex and age were determined by examining wolves at the capture site (*n*_males_ = 14, n_females_ = 12). Age was determined by assessing the dental pattern, and individuals were classified as subadults (1 to 2 yr, *n* = 16) or adults (≥2 yr, *n* = 10) (Gipson et al. 2000). Wolves were also classified as pack members (*n* = 19) and nonpack members (dispersers and lone wolves, *n* = 7). Pack membership was determined by evaluating movements in relation to confirmed den and rendezvous sites. These sites were identified through standardized howling surveys (Llaneza et al. 2014; Palacios et al. 2016) and supplemented with visual observations of both collared and noncollared individuals. Wolves were classified as pack members when they were repeatedly observed, either through GPS relocations or direct sightings, using or approaching the same den or rendezvous site where pups or other pack members had been independently confirmed. In contrast, wolves were classified as nonpack members when they showed no repeated association with any confirmed den or rendezvous site, were never observed in the vicinity of pups or pack groups, and displayed movement patterns consistent with dispersal or solitary ranging. We further assigned GPS locations to 3 significant periods in the biological cycle of wolves, defined by reproduction: mating season (February to April), breeding season (May to October) and nonreproductive period (November to January) (Álvares 2011).

### Wolf movement metrics

We calculated 3 metrics of wolf movement based on 24-h tracks: daily distances traveled, net displacement and straightness index. Together, these metrics provide a multifaceted and complementary description of wolf movement at daily scales, linked to key attributes of their ecology such as foraging, territory patrolling and maintenance, and navigation efficiency across the landscape. Daily distances were estimated as the sum of step lengths during a 24-h track, where step length was the distance between 2-h consecutive GPS positions. Net displacement was defined and estimated as the Euclidean distance from the first to the last position recorded within a 24h-track. Daily tracks were defined between 16:00 and 14:00 of the following day based on the activity patterns of the wolf population in the region, as it properly captures circadian rhythms between main daily resting periods (Ferreiro-Arias et al. 2024). Straightness index was calculated as the ratio between net displacement and daily distance of each 24-h track. The straightness index is a proxy of path tortuosity, defined as the degree to which a movement path deviates from a straight-line (Batschelet 1981). Straightness index values range from 0, indicating high tortuosity in movement during the trajectory, to 1, depicting a fully straight and directional movement. For each day, we further estimated a measure of track completeness by calculating the proportion of missing locations from the total locations that could be recorded in a day (12 locations at most) and keeping only tracks with >90% of completeness (≤ 1 missing location) for further analysis. Subsequently, we calculated a set of summary statistics including mean, median, standard deviation (SD), min, max, first and third quantiles and interquartile range (IQR) of daily distances, net displacement and straightness index for the daily trajectories of each wolf.

### Spatial data and covariates

To extract information on predictors related to anthropogenic disturbance, trophic resources, landscape attributes and refuge cover (Table 1), we first created a 1-km buffer around each daily track, which approximately is equal to the mean of the step lengths (mean ± SD: 0.8 ± 1.4 km). We employed these buffers as “influence areas” of each predictor in order to extract the values of each layer and calculate the average value of each variable. This resulted in a single daily value for each predictor variable per wolf movement track, representing the average environmental conditions experienced during that 24-h period.

**Table 1:** Description of variables used to explore differences in wolf daily movements.

Predictor	Factor	Description
**Age (Categorical)**	Intrinsic	Age class of the individual: Subadult (<2 yr) or Adult (≥2 yr).
**Sex (Categorical)**	Intrinsic	Sex class of the individual: Female or Male.
**Social status (Categorical)**	Intrinsic	Group affiliation of the individual: Pack member (belonging to a pack) or nonpack member (disperser or solitary individual).
**Reproductive period (Categorical)**	Intrinsic	Seasons of reproduction for Iberian wolves: Mating (Feb-Apr), Reproductive (May-Oct), and Nonreproductive (Nov-Jan)
**Human population density (Continuous, inhab./km^2^)**	Anthropogenic	Number of inhabitants per km^2^ within a 1 km^2^ buffer around daily track.
**Human settlement density (Continuous, settlements/km^2^)**	Anthropogenic	Density of human settlements (buildings or clusters) per km^2^ within a 1 km^2^ buffer around daily track.
**Primary road density (Continuous, km/km^2^)**	Anthropogenic	Length (km) of major paved roads (eg, highways) per km^2^ within a 1 km^2^ buffer around daily track.
**Second road density (Continuous, km/km^2^)**	Anthropogenic	Length (km) of minor paved roads (eg, local roads) per km^2^ within a 1 km^2^ buffer around daily track.
**Diet category (Categorical)**	Trophic resources	Dominant prey type in the local area, based on regional diet analysis: Livestock, wild prey (mostly roe deer and wild boar), or Free-ranging horses.
**Terrain Ruggedness Index (Continuous, unitless)**	Landscape attribute	Mean topographic heterogeneity index quantifying the elevation difference between adjacent cells within a 1 km^2^ buffer around daily track.
**Percentage of cover (Continuous, percentage)**	Refuge cover	Proportion (%) of the 1 km^2^ buffer around daily track classified as vegetation that could serve as refuge for wolves.
**Patch Density (Continuous, patches/km^2^)**	Refuge cover	Number of refuge patches per km^2^ within a 1 km^2^ buffer around daily track.
**Mean patch size (Continuous, km^2^)**	Refuge cover	Average area of refuge patches within a 1 km^2^ buffer around daily track.
**Mean Cohesion Index (Continuous, unitless)**	Refuge cover	Average physical connectedness of refuge patches within a 1 km^2^ buffer around daily track.
**Mean Fractal Dimension (Continuous, unitless)**	Refuge cover	Mean shape complexity of refuge patches within a 1 km^2^ buffer around daily track.

We assessed the impact of anthropogenic disturbance by analyzing different proxies of human infrastructure encroachment, as density of settlements and paved roads, and human activity (human population density) that might affect wolf movement (Additional File 2, Table S2). To obtain values for human settlements and population density, we utilized data on >30,000 individual population entities in Galicia, including their population sizes from 2010 obtained from the Spatial Data Infrastructure of Galicia (IDEG, http://mapas.xunta.gal/ideg). We used these population entity locations to estimate the density of human settlements, applying the “*Kernel Density*” tool in ArcGIS Pro (Environmental Systems Research Institute 2023), weighting each population entity location based on its population density. For paved roads, we initially classified roads obtained from IDEG into primary and secondary categories based on traffic volume, fencing, and lane numbers (Dennehy et al. 2021), and then calculated the density of these road categories within buffers around wolf daily trajectories.

We extracted Terrain Ruggedness Index (TRI) values for each daily trajectory, which measures the variation in terrain elevation within a given area (Riley et al. 1999). Differences in terrain ruggedness can influence the daily movements of wolves, as more rugged terrain may pose resistance and higher energy costs to wolf movement (Whittington et al. 2004). Furthermore, daily movements of wolves are expected to change for different categories of prey availability, the spatial distribution and density of prey, and the exposure to humans associated with predation on the most abundant prey type (López-Bao et al. 2013; Lagos and Bárcena 2018). Hence, we examined whether daily wolf movements are influenced by their primary prey type, classifying individual wolves into 3 categories according to the main prey available in the region: free-ranging horses, other livestock, and wild prey (roe deer and wild boar) (Additional File 2, Table S2). This classification was based on a spatial overlay of our wolf monitoring data with established geographic patterns of wolf diet derived from scat analysis in the same region. Specifically, we assigned each collared individual to the dominant prey category (livestock, wild prey, or horses) documented for its local area in prior studies of wolf trophic ecology (López-Bao et al. 2013; Lázaro-Fuentes 2014). This approach provided a landscape-scale proxy for prey availability, circumventing the lack of systematic, fine-scale abundance data for wild ungulates across our entire study area.

We estimated the availability and structural characteristics of refuge cover to test their influence on wolf movement (Additional File 2, Table S2). We defined refuge cover as the area occupied by vegetation patches that could provide shelter for wolves (Llaneza et al. 2016). We extracted patches of plantations, forest, dense scrubland and other cover types that provide shelter for wolves from land use digital maps (SIOSE National Technical Team 2022). Then, we rasterized the spatial layers with a resolution of 10 × 10 m and calculated the availability and characteristics of refuge patches. Different attributes of vegetation patches, such as size, connectivity, and human intervention can significantly influence refuge quality (Llaneza et al. 2016, 2018; Grilo et al. 2019; van den Bosch et al. 2023). Hence, we retrieved different landscape metrics to characterize refuge cover in terms of both its total extent (quantity) and its spatial configuration (quality). We extracted 2 metrics at the patch level: Fractal Dimension Index (FDI) and refuge patch size. We calculated the FDI as a proxy of human influence on patches of refuge cover (Imre and Bogaert 2004; Bista et al. 2021). FDI describes the shape complexity of each habitat patch based on perimeter-area relationships ranging between 1 and 2, with values close to 1 for simple geometric perimeters and values closer to 2 for highly convoluted patch boundaries. Natural patches of vegetation usually have more complex structures and irregular shapes and are positively selected by wolves (van den Bosch et al. 2023), compared with human-modified vegetation patches such as monospecific forest plantations or croplands which feature simple polygonal shapes, usually rectangles (Imre and Bogaert 2004; Bista et al. 2021). Refuge patch size was defined and calculated as the area covered by each patch of vegetation potentially acting as a refuge for wolves. We measured the size of all refuge patches within each influence area and calculated the mean value. Additionally, we computed 3 landscape-level metrics: Patch Cohesion Index (PCI), refuge cover and refuge patch density. We estimated PCI as a proxy of connectivity between refuge patches. PCI ranges from −1 depicting a totally isolated patch to 1 indicating highly connected refuge patches (Schumaker 1996). Refuge cover was calculated as the percentage of the buffer area occupied by vegetation classified as potential refuge for wolves. Refuge patch density was determined by dividing the number of refuge patches within the buffer by the total buffer area. All refuge cover metrics were calculated using the “*landscapemetrics*” package (Hesselbarth et al. 2019).

### Modeling wolf movement

We modeled wolf movement employing Bayesian Regression Models (hereafter, BRMs). Instead of constructing multiple competing models with different subsets of predictors and performing model selection based on information criteria (eg, AIC or BIC), we adopted a hypothesis-driven approach for modeling. We built a single, comprehensive model with fixed and random structure defined a priori by our ecological hypotheses (Additional File 2, Table S2). Prior to modeling, we normalized daily distances and net displacement through a square-root transformation as this approach significantly improved the fit of the model and the distribution of residuals. For models using daily distance or net displacements as the response variables, we fitted a Gaussian error distribution, while for modeling the straightness index, we fitted a zero-inflated Beta error distribution. We incorporated as predictors age, sex, social status, reproductive period, diet category, and the spatial predictors related to anthropogenic disturbance, refuge cover and landscape attributes (Table 1, Additional File 2 Table S2). To avoid multicollinearity, we assessed the correlation structure among predictors and VIF values of our model. Due to the high correlation between various refuge cover metrics (*r* ≥ 0.7), we used a Principal Component Analysis (PCA) with the “*FactoMineR*” package (Lê et al. 2008) (Additional File 1, Figure S1a and b). The first 2 dimensions of the PCA explained 83.4% of the variance, with the first dimension of the PCA accounting for 67.9% and the second for 15.5% (Additional File 1, Figure S1a). The first dimension was positively influenced by the percentage of refuge cover within influence areas, the cohesion among refuge patches and, to a lesser extent, patch size, and negatively influenced by patch density (Additional File 1, Supp. Figure S1b). The second dimension was characterized by the main positive contributions from the FDI and negative from patch density (Additional File 1, Figure S1b). The first dimension distinguished landscape samples with extensive and cohesive refuge cover from those with fragmented, sparse vegetation cover. The second dimension differentiated refuge cover made of patches with complex, irregular shapes from denser cover made of patches with geometric-like shapes (Additional File 1, Figure S1a). Consequently, we used the coordinates of the first 2 principal components as predictors in our models. The model also included a priori defined interaction terms to explore potential synergistic and buffering effects, specifically between sex and social status with reproductive period, human population density and human settlement density, primary and secondary road densities and refuge, and both refuge dimensions (Additional File 2, Table S2). All continuous predictors were scaled and centered around zero with a SD equal to 1 before model fitting. To control for the lack of independence in repeated measurements for the same individuals, we included wolf identity as a random intercept (Hebblewhite and Merrill 2008). We did not specify a nested effect of wolf identity within wolf pack because only 2 wolves were members of the same pack (Hebblewhite and Merrill 2008).

To avoid temporal autocorrelation inherent to movement data, we subsampled two thirds of our dataset. We randomly selected observations while maintaining the proportion of data obtained from each individual and each month, thereby preventing bias. We chose this subsampling approach over alternatives such as incorporating autoregressive terms, because it directly breaks the temporal sequence causing autocorrelation. This sacrifices a fraction of observations while retaining sufficient power for predictors, interactions, and random effects, without adding unnecessary analytical complexity. To address spatial autocorrelation, we created a 1 × 1 km grid across the study area, assigning a unique identifier to each cell. Each daily trajectory was then linked to a specific grid cell using the mid longitude and latitude values and the identity of each grid cell was subsequently included as a random effect in the model, hereafter referred as fine-scale random spatial effects (Dormann et al. 2007). These random spatial effects capture unmeasured local variation in wolf daily movement patterns associated with the specific location of each daily trajectory, accounting for spatial heterogeneity not explained by the fixed effects. We ran the BRMs with 4 MCMC chains with 4,000 iterations each, applying a warm up of 2,000 iterations. We specified weakly informative priors using a normal distribution *N*(*0,10*) for the intercept and *N*(*0,1*) for slope coefficients (Lemoine 2019). Bayesian regression models were fitted employing the “*brms*” package (Bürkner 2021) in R v.4.4.0 statistical software (R Core Team 2023). Chain convergence was checked by the R-hat diagnostic (R-hat ≈ 1). Temporal autocorrelation was tested using autocorrelation using the *acf()* function from “*stats*” package (R Core Team 2023). Spatial autocorrelation in model residuals was tested by calculating Moran's I with “*DHARMa’* package (Hartig and Hartig 2017). Conditional and Marginal *R*^2^ were computed using the “*performance*” package (Lüdecke et al. 2021). Model diagnosis and marginal effects were plotted using the “*brms*”, “*ggplot2*” and “*sjPlot*” packages (Wickham 2016; Bürkner 2021; Lüdecke 2022).

### Variable importance

We estimated the Probability of Direction (PD) using the “*bayestestR*” package (Makowski et al. 2019) as a measure of the presence of every predictor on the response variable tested. The PD ranges from 0.5 to 1.0 and represents the level of confidence with which an effect is observed to occur in a specific direction, where PD > 0.9 was considered a credible threshold of effect direction (Makowski et al. 2019). We only considered significant predictors when their 95% credible intervals did not cross zero. Additionally, we assessed the relative importance of the predictors included in our models with a variance partitioning approach. Initially, we estimated a pseudo-*R*^2^ by squaring the correlation coefficient between the median posterior prediction of our model and the observed movement metrics (ie, daily distance, net displacement and straightness index). Next, we conducted this process iteratively for each predictor while holding it at its mean value for continuous variables. For categorical variables, we systematically replaced all levels with the same level, and for random effect variables, we substituted all random effect levels with a new level that did not exist in the dataset. By subtracting the resulting pseudo-*R*^2^ values of each predictor from the initial pseudo-*R*^2^, we derived a measure of variable importance (average pseudo-*R*^2^ for the tested level in categorical variables). To represent the relative importance of predictors, we expressed their contribution to the pseudo-*R*^2^ as a percentage (see Ferreiro-Arias et al. 2025 for a detailed description of this approach).

## Results

### Daily distances

**Figure 2:**
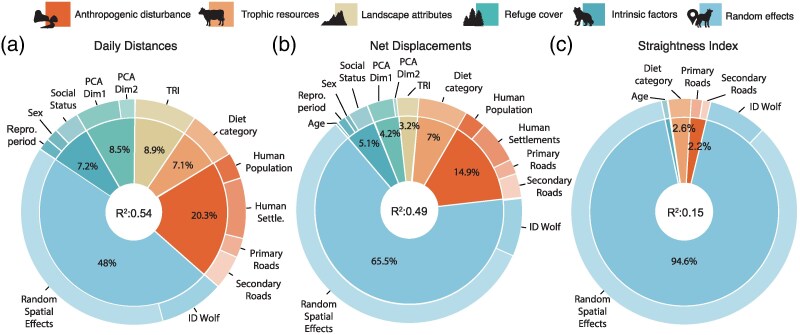
Ranked predictor contributions to the total variance captured by the Bayesian Regression Models explaining a) daily distances, b) net displacements, and c) straightness of wolf daily movements. Donut plots show the percentage contribution of each major group of predictors including anthropogenic disturbance, trophic resources, landscape attributes, refuge cover, intrinsic factors, and random effects, to the total pseudo-R^2^, indicated at the center of each plot.

**Figure 3:**
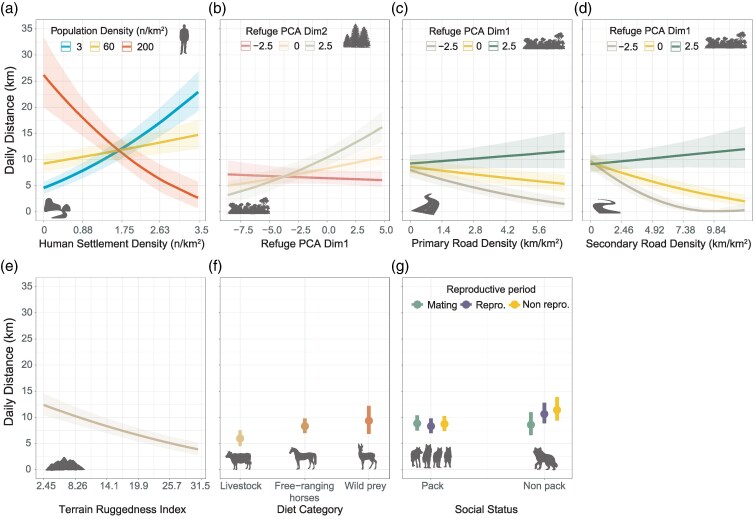
Marginal effects of the main predictors explaining differences in daily distances traveled by wolves: a) the interaction between human population density and human settlement density; b) the interaction between the first and second dimensions of refuge cover PCA; c) the interactions between primary road density and d) secondary road density with the first dimension of refuge cover PCA; and the effects of e) terrain ruggedness, f) diet category, and g) the interaction of social status with reproductive periods. Values of predictors within figure legends were selected according to the first quartile and third quartile of each variable. Shaded contours depict the 95% confidence intervals.

Overall, based on a 2-h sampling interval, wolves traveled an average (±SD) distance of 9.0 (± 5.8 km) km per day. Minimum and maximum distance traveled during 24-h periods was 0.02 km and 39.4 km, respectively (Table 2). The BRM explaining the factors influencing daily distances traveled by wolves showed a Conditional *R*^2^ of 0.54 (95% CI [0.51, 0.57]) and a Marginal *R*^2^ of 0.27 (95% CI [0.22, 0.32]). A significant portion of the explained variance (48%) was attributed to random effects, particularly to fine-scale random spatial effects (38.6%), followed by the wolf identity (9.5%) (Fig. 2a). Anthropogenic disturbance proxies accounted together for 20.3% of the variance and exhibited significant effects (Additional File 2, Table S3), with human settlement density (8.7%) and secondary road density (4.98%) being the main contributors (Fig. 2a). We found a significant interaction between settlement and populations density (Table S3), showing that wolves traveled longer daily distances when human population density was either low and highly dispersed (ie, high human settlement density) or high but concentrated. Wolves reduced considerably daily distances traveled when human population density was either high and highly dispersed or low and concentrated (Fig. 3a).

**Table 2:** Summary statistics of different wolf movement metrics.

Metric	Min	Q1	Median	Mean	Q3	Max	IQR
**Daily distance**	0.02	4.61	7.9	8.98	12.37	39.43	7.75
**Net displacement**	0.001	0.92	2.89	3.83	5.5	30.17	2.61
**Straightness Index**	0.001	0.18	0.41	0.44	0.66	1	0.48

We found that refuge cover had a significant positive effect on daily distances (Additional File 2, Table S3), accounting for ∼8.5% of the variance, with the first PCA dimensions contributing ca. 3 quarters to this variance. Both dimensions of refuge cover PCA indicate that wolves increased their movements when extensive and highly cohesive refuge cover is available. We further found significant effects of the interaction between both PCA axis with paved road densities. Highly extensive and cohesive refuge cover attenuated the negative effect of primary and secondary road density on wolf daily distances (Fig. 3c, d). Terrain ruggedness had a significant negative effect on daily distances and explained 8.9% of the variance (Additional File 2, Table S3). Diet category explained 7.1% of the variance, with significant differences found for wolves that feed on livestock, which traveled significantly shorter daily distances than those feeding on free-ranging horses or wild prey (Fig. 3f, Additional File 2, Table S3). Intrinsic factors accounted for 7.2%, with social status contributing the most (3.76%), but none of them exhibited a significant effect, except the interaction between social status and reproductive period (Additional File 2, Table S3). Pack members did not exhibit differences in daily distances covered among different reproductive periods. In contrast, nonpack members traveled longer distances per day during reproductive and nonreproductive periods compared with the mating period (Fig. 3g, Additional File 2, Table S3).

### Net displacement

**Figure 4:**
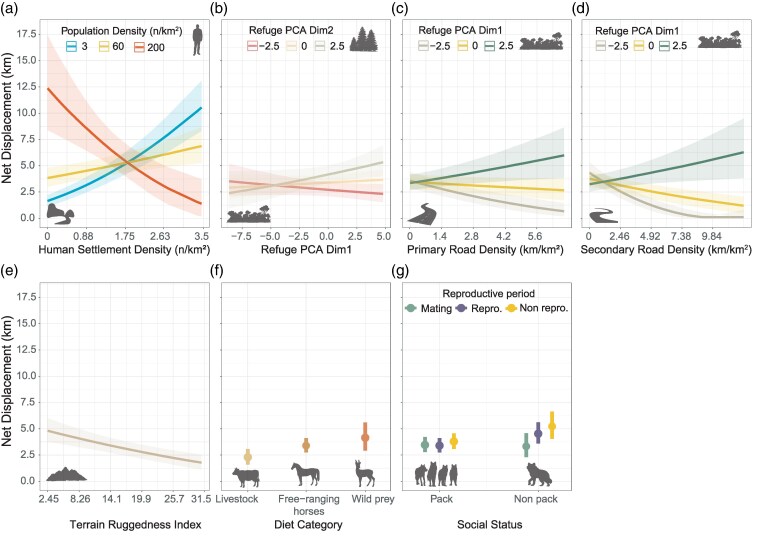
Marginal effects of the main predictors explaining differences in net displacements performed by wolves: a) the interaction between human population density and human settlement density; b) the interaction between the first and second dimensions of refuge cover PCA; c) the interactions between primary road density and d) secondary road density with the first dimension of refuge cover PCA; and the effects of e) terrain ruggedness, f) diet category, and g) the interaction of social status with reproductive periods. Values of predictors within figure legends were selected according to the first quartile and third quartile of each variable. Shaded contours depict the 95% confidence intervals.

The mean (±SD) net displacement over 24-h cycles was 3.83 km (± 3.55 km). Minimum and maximum net displacements within 24-h period were 0.001 and 30.17 km (Table 2). Our results indicate that net displacements and daily distances traveled were affected by the predictors in a very similar way (Figs. 3 and 4). The BRM explaining the factors influencing net displacements showed a Conditional *R*^2^ of 0.49 (95% CI [0.46, 0.52]) and a Marginal *R*^2^ of 0.18 (95% CI [0.14, 0.23]). As with daily distances, most of the explained variance (65.5%) was attributed to random effects, with fine-scale random spatial effects capturing 57.2%, followed by wolf identity (8.4%) (Fig. 2b).

Anthropogenic disturbance proxies captured 14.9% of variance (Fig. 2b) with settlement density (6.11%) accounting for the larger amount of this explained variance (Fig. 2b). We found significant effects of settlement and population densities, including its interaction (Additional File 2, Supp. Table S4). Wolves increased their net displacements when the human population density was low and highly dispersed, while they reduced considerably their net displacements when human population density was high and highly dispersed (Fig. 4a). Contrary to daily distances, we did not observe significant individual effects of primary road density or the first refuge PCA dimension on net displacement, but their interaction was statistically significant (Additional File 2, Table S4). Secondary roads showed significant individual effects and with its interaction with first refuge PCA dimension (Additional File 2, Table S4). In this sense, we observed a significant buffering effect of highly extensive and cohesive refuge cover on the density of primary and secondary roads (Fig. 4c, d), whereas both types of roads negatively impacted the net displacements of wolves when the available refuge cover was lower and less cohesive (Fig. 4c, d).

Both dimensions of refuge cover PCA captured 4.2% of the total variance explained (Fig. 2b), and only the second dimension showed a significant positive effect (Table S4). We found a significant interaction of both dimensions of the refuge cover PCA showing that wolves increased their net displacements when extensive and highly cohesive natural refuge cover with complex shapes was available (Fig. 4b, Additional File 2, Table S4). Conversely, wolves slightly curtailed their displacements when refuge cover had low fractal values and a high density of refuge patches, despite a wide availability and cohesion of refuge cover (Fig. 4b).

Terrain ruggedness also had a significant negative effect on net displacements, accounting for the 3.2% of explained variance (Fig. 4e, Additional File 2, Table S4). The class of dominant available prey type was the fixed predictor that captured the highest percentage of variance (7%) and wolves feeding mostly on livestock exhibited significantly shorter net displacements compared with those using areas where the density of free-ranging horses or wild prey was high (Fig. 4f, Additional File 2, Table S4). Intrinsic factors explained the 5.1%, with most of this variance captured by the social status of the individual (2.9%) and the reproductive periods (1.2%) which were the only intrinsic factors showing a significant interaction effect (Additional File 2, Table S4). Pack members did not show differences in net movements across different reproductive periods, while nonpack members exhibited longer net displacements during both reproductive and nonreproductive periods compared with the mating season. Finally, net displacements of nonpack members were higher than pack members during reproductive and nonreproductive periods (Fig. 4g).

### Straightness index

**Figure 5:**
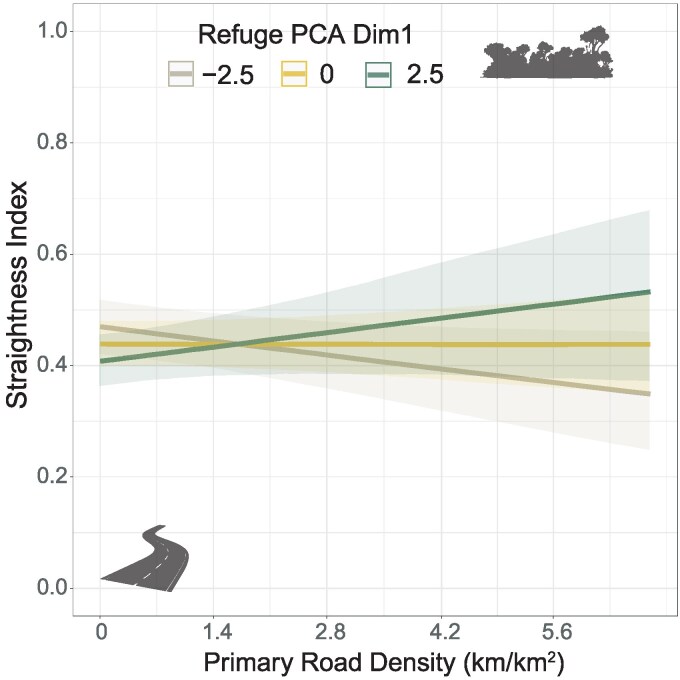
Marginal effect of the interaction between primary road density and the first dimension of refuge cover PCA explaining differences in tortuosity of wolf daily trajectories. Values of the first dimension of refuge cover PCA were selected according to the first quartile and third quartile of the variable. Shaded contours depict the 95% confidence intervals.

The mean (±SD) straightness index of daily wolf movement was 0.44 (± 0.28). Minimum and maximum straightness index for 24-h trajectories were 0 and 0.99 respectively (Table 2). The BRM explaining the factors influencing straightness in daily wolf movements showed a Conditional *R*^2^ of 0.15 (95% CI [0.12, 0.19]) and a Marginal *R*^2^ of 0.05 (95% CI [0.03, 0.07]). Random effects captured 94.6% of the explained variance, most of which was accounted for random spatial effects (88.1%), followed by wolf identity (6.5%) (Fig. 2c). Anthropogenic disturbance proxies explained 2.2% of variance in straightness index, including the effects of primary and secondary road densities (1.82% and 0.38%, respectively). The availability of dominant prey types explained 2.6% of the variance captured whereas intrinsic factors, mostly age, explained 0.6%. Contrary to the other movement metrics, we found only a significant effect of the interaction between primary road density and the first dimension of refuge cover PCA (Additional File 2, Table S5). When refuge availability was low, the tortuosity of wolf daily movements increased with the density of higher primary roads. Under these same circumstances wolves maintained straighter movements if refuge patches were extensive and cohesive (Fig. 5).

### Model diagnosis

R-hat convergence diagnostic showed values equal to 1 for all coefficients, indicating successful convergence of the MCMC chains for all models (Additional File 2, Tables S3 to 5). Model posterior predictive checks closely matched all observed movement metrics, indicating a good fit to the data (Additional File 1, Figures S3 to S5). Model residuals did not exhibit any temporal or spatial autocorrelation (Additional File 1, Figures S3 to S5).

## Discussion

Our study contributes to the understanding of the factors influencing daily movements of a top-predator in human-dominated landscapes, modulated by anthropogenic disturbance, refuge cover, terrain ruggedness, main prey consumed, and intrinsic factors. Our analysis of variance partitioning revealed that the most influential factor driving reduction of distances traveled by wolves was anthropogenic disturbance, followed by refuge cover, terrain ruggedness, the type of prey consumed (ie, wild prey vs. domestic ungulates), and intrinsic factors. Daily distance traveled and net displacement showed highly convergent responses to these predictors, indicating that wolves adjusted not only how far they moved in total but also how far they moved away from their starting location in broadly similar ways. In contrast, path tortuosity estimated by the straightness index diverged from these patterns and was only significantly affected by the interaction between refuge cover and primary road density. How direct wolf paths are seems to depend upon specific constraints linked to navigation around high-risk linear features and the availability of protective cover. Overall, we found that different descriptors of movement respond to different behavioral tradeoffs, with tortuosity likely linked to fine-scale decision-making related to risk avoidance, foraging, or terrain complexity that were not included in our models.

In line with previous studies highlighting carnivore movement reductions in areas with high human footprint (Tucker et al. 2018, 2023; Lamb et al. 2020; Barceló et al. 2025; Morant et al. 2025), we found that the most important factor driving movement reduction was anthropogenic disturbance. Wolves increased movements in areas of low population density and dispersed settlements, and reduced them in areas with high or highly dispersed populations. This pattern suggests that wolves exploit human-sparse areas to move more freely across the landscape, likely because the probability of encountering people, infrastructure, or human-related risks is low. In contrast, dense or spatially concentrated human settlements appear to constrain wolf mobility, prompting shorter or more localized movements as wolves attempt to minimize detection and avoid risky areas. These findings reinforce a broader pattern documented in many carnivore systems globally, whereby anthropogenic landscapes create a human-mediated landscape of “fear” that shapes movement across multiple scales (Odden et al. 2014; Gaynor et al. 2018; Lamb et al. 2020; Mills et al. 2023). Wolves in our study landscape appear to conform to these general predictions of risk-sensitive movement theory where individuals reduce displacement and adapt their movements when perceived risk increases, similar to responses observed in other large predators such as bears, lions or leopards living near human settlements (Odden et al. 2014; Whittington et al. 2022; Mills et al. 2023).

Besides human settlements and population, paved roads also constrained daily distances and net displacements and led to more tortuous paths. Roads, especially primary ones, can serve as both physical barriers and sources of mortality risk for top-predators (Quintana et al. 2022; Denneboom et al. 2024). Nevertheless, several studies suggest that paved roads with high traffic volumes are avoided by large carnivores rather than acting as facilitators of long exploratory and foraging movements, as observed in less anthropized landscapes (Blanco et al. 2005; Dickie et al. 2017; DeMars and Boutin 2018; Kautz et al. 2021; Denneboom et al. 2024). In our study area, wolves inhabit one of the most exposed regions of their distribution range to this type of human infrastructure (Ceia-Hasse et al. 2017). As the density of primary roads increased, wolves increased the tortuosity of their trajectories, which potentially indicates that they shift the direction of their movements when the permeability of the landscapes is reduced due to roads. However, this effect was not significant for secondary paved roads, which is in line with previous findings indicating increases in the tortuosity of carnivore movement paths depending on the level of human activity associated to linear infrastructures (Whittington et al. 2004; Barceló et al. 2025). This behavioral adjustment mirrors global patterns in which linear infrastructure restructures animal movement by altering landscape connectivity, modifying decision-making at junctions, and imposing asymmetric permeability across landscapes (Whittington et al. 2004, 2022; Thorsen et al. 2022). The increased tortuosity near primary roads is consistent with a general response of large mammals navigating fragmented habitats, reflecting a general tradeoff between movement efficiency and avoidance of predictable mortality sources (Dennehy et al. 2021; Thorsen et al. 2022; Whittington et al. 2022). Despite the potential limitation for movement, recent studies evidenced that carnivores tend to increase their nocturnal activity as they get closer to paved roads and crossing events when levels of traffic is lower (Dennehy et al. 2021; Kautz et al. 2021; Ferreiro-Arias et al. 2024). Hence, although carnivores may adapt their trajectories and limit their movements to avoid primary paved roads during daytime, temporal partitioning with human activity may weaken the potentially negative effects of roads on landscape permeability for top-predators.

We found that the extent and cohesion of vegetation cover acting as a refuge had a significant buffering effect over the negative effect on movement impeded by these types of infrastructures. The possibility that vegetation cover attenuates the negative effects of roads highlights its important role in allowing persistence of carnivores under anthropogenic disturbance (Grilo et al. 2015; Llaneza et al. 2016; Sazatornil et al. 2016; Grilo et al. 2019; Ferreiro-Arias et al. 2024; Jansson et al. 2024). Besides this, our findings suggest that not only the availability of large extents of refuge cover, but also its spatial configuration, can influence how top-predators navigate landscapes fragmented by humans. While the distances and tortuosity of their trajectories are primarily influenced by quantitative aspects of cover (ie, extent of refuge cover), its qualitative attributes (ie, spatial configuration) also shape daily distances and net displacements. During the past decade, most studies exploring how vegetation cover influences different aspects of the spatial ecology of these carnivores have focused on the extent of refuge cover available, neglecting the role of the spatial configuration and other qualitative components of such resource (Llaneza et al. 2012, 2016; Grilo et al. 2019; Bista et al. 2021; Ferreiro-Arias et al. 2024; Morant et al. 2025). Our results show how different attributes of the distribution of vegetation cover, such as cohesion, size, density and shape of refuge patches, contribute to keep landscape permeability to carnivores and help them to cope with anthropogenic disturbance.

Within human-dominated landscapes, a key factor influencing the movements of top-predators is prey availability (Valeix et al. 2010; Petroelje et al. 2019; Barceló et al. 2025). In line with our hypothesis, for individuals foraging on livestock we detected reductions in daily distances and net displacements compared with individuals using areas where free-ranging horses and wild ungulates, such as roe deer and wild boar, were the main trophic resource. These patterns parallel general predictions from optimal foraging and central-place foraging theory, wherein animals adjust travel distances based on the spatial predictability and aggregation of resources (Orians and Pearson 1979). The reduction in movement associated with differences in the dominant prey type is likely related to differences in the spatio-temporal availability of livestock, which often concentrates in large numbers in fixed locations, compared with the more scattered distribution of wild ungulates or the extensive husbandry regime of free-ranging horses in our study area (López-Bao et al. 2013; Lagos and Bárcena 2018). Considering the development of spatial memory while choosing among diverse foraging strategies, carnivores can optimize their movements in the presence of fixed locations of abundant trophic resources by adopting, for example, central-place foraging patterns (Valeix et al. 2010; López-Bao et al. 2011; Gurarie et al. 2022; Iorio-Merlo et al. 2022). This strategy would allow wolves to travel between their dens or resting sites and areas where livestock is concentrated and vulnerable to predation, reducing the number and length of foraging trips.

Lastly, intrinsic factors did not exert much influence on daily movements and only social status emerged as a consistent factor influencing distances traveled. The influence of social status suggests the existence of a behavioral polymorphism in movement strategies driven by sociality. In group-living carnivores like some canids and lions, the territorial and central-place based movement patterns by pack or pride members, contrasts sharply with the nomadic movements of nongroup members (Mancinelli et al. 2019; Ylitalo et al. 2021; Jansson et al. 2024; Benson et al. 2025). The observation that pack members exhibited shorter movements during the reproductive period compared with nonpack members, could be related to homesite-based movements likely departing and returning to den or resting sites and linked to the maintenance of their territories (Rio-Maior et al. 2018; Ylitalo et al. 2021). Round-trips of pack members may result in shorter net displacements, particularly during reproductive periods when the need to nurse and feed pups and homesite attendance limit parent movements (Mech and Boitani 2003; Rio-Maior et al. 2018; Ylitalo et al. 2021). In contrast, nonpack members tend to be nomadic individuals not attached to stable territories (Fuller et al. 2003), which likely induce longer net displacements and daily distances compared with pack-members. Contrary to our hypothesis, we found that nonpack members increased their movement during the reproductive and nonreproductive periods compared with the mating period, when looking for new mates would potentially increase the distance traveled by these individuals (Mech and Boitani 2003). Nonpack members are known to exhibit behavioral shifts to avoid aggressive encounters with dominant pack members resulting in an increase of diurnal activity (Mancinelli 2018), avoidance of scent-marking and howling within pack territories (Mech and Boitani 2003) or moving along the periphery of occupied territories by wolf packs (Kirilyuk et al. 2020). An increase in distances traveled by nonpack members during the reproductive period may reflect the need of resources outside pack territories where and when surveillance, marking and aggressive behavior by pack members is more frequent and intense (Mech and Boitani 2003).

Our study adds to the growing body of evidence that carnivores, and particularly wolves, exhibit behaviors that reflect an apparent adaptation of their spatial and behavioral ecology to human-dominated landscapes (Eggermann et al. 2011; Llaneza et al. 2012, 2016; Sazatornil et al. 2016; Dennehy et al. 2021; Martínez-Abraín et al. 2023; Ferreiro-Arias et al. 2024). Given the intense persecution by humans that wolves have faced over the centuries (Rico and Torrente 2000), these behavioral strategies could reflect wolf adaptations to avoid humans. In landscapes offering abundant trophic resources but fraught with high human-induced mortality risks, traveling long distances may be counterproductive for carnivores as it increases the likelihood of encounters with humans. By positioning wolves as part of a broader pattern of adaptive movement strategies that shape carnivore persistence in increasingly human-dominated ecosystems, our results further reinforce emerging principles showing that large carnivores mitigate human pressures through a combination of spatial, temporal, and behavioral adjustments (Odden et al. 2014; Tucker et al. 2018, 2023; Lamb et al. 2020; Mills et al. 2023; Ferreiro-Arias et al. 2024). Further research is needed to investigate whether these behavioral strategies are adaptive.

## Supplementary Material

arag037_Supplementary_Data

## Data Availability

Analyses reported in this article can be reproduced using the data provided by Ferreiro-Arias et al (2026). Raw data supporting our findings of this study are available on request from *A.RE.NA. Asesores en Recursos Naturales*, S.L. Raw data is not publicly available as it includes information of location of wolves and packs, a protected species under current Spanish legislation (Spanish Royal Decree 139/2011 and Ministerial Order TED/980/2021).
