# Supplementary material for: Human disturbance, preys and refuge cover shape top predator movements in anthropogenic landscapes
Source: Behav Ecol. 2026 Apr 13;37(3):arag037. doi: 10.1093/beheco/arag037 (PMC13121897; doi:10.1093/beheco/arag037)
Supplement: arag037_Supplementary_Data [file arag037_supplementary_data.zip › CLEAN_Additional_File_1_Supp_Figures_Wolf_Movement_Manuscript.docx]

**Supplementary Figures.**

**
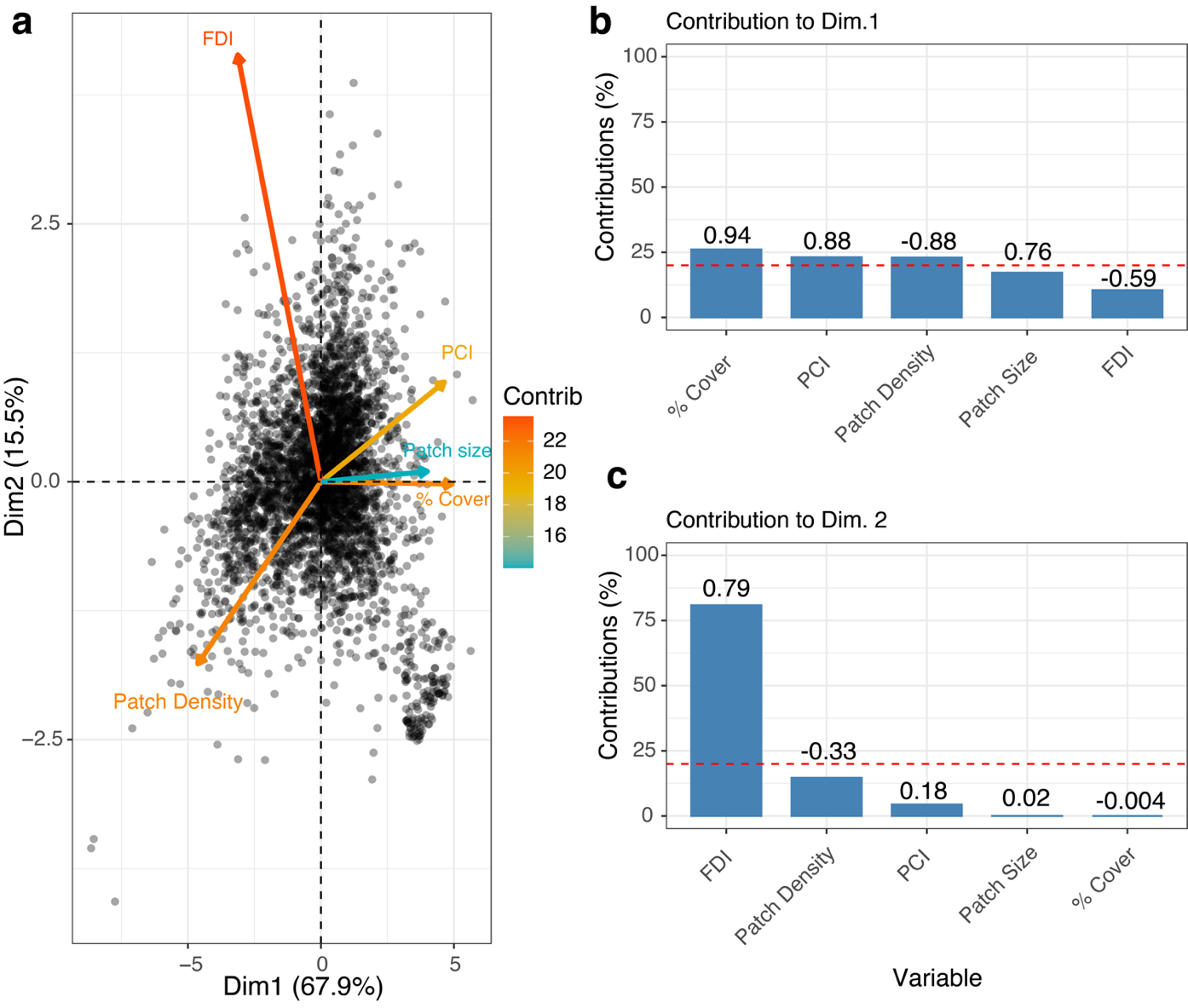
­­­­­Supplementary Figure 1.** Principal Component Analysis (PCA) of refuge cover variables. (a) Scatterplot of the first two principal components (PC1 and PC2), showing the percentage of variance explained by each component. Contribution of the refuge cover variables to PC1 (b) and PC2 (c), including loadings of each variable on each dimension. The first dimension (PC1) was positively influenced by the percentage of refuge cover within influence areas, the cohesion among refuge patches, and, to a lesser extent, patch size, and negatively influenced by patch density. The second dimension (PC2) was characterized by the main positive contributions from the Fractal Dimension Index and a negative contribution from patch density.

**
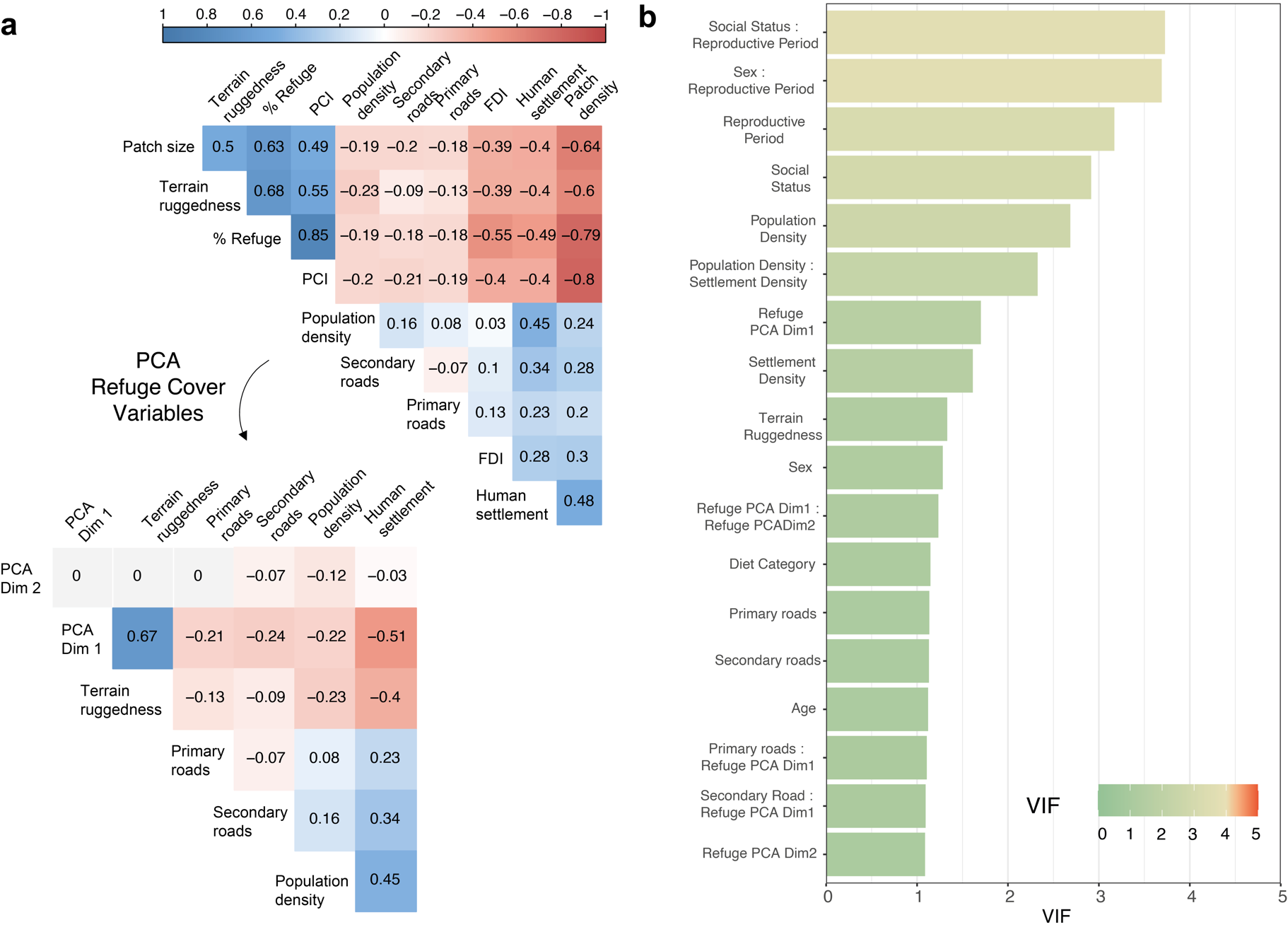
Supplementary Figure 2.** Multicollinearity analysis of predictors. Panel a shows Pearson correlation coefficients (r) for pairwise comparisons among all predictors and after reducing the dimensionality of refuge cover variables by including the coordinates of the first and second dimensions of the refuge cover PCA. Panel b depicts VIF values included in Bayesian Regression Models.

**
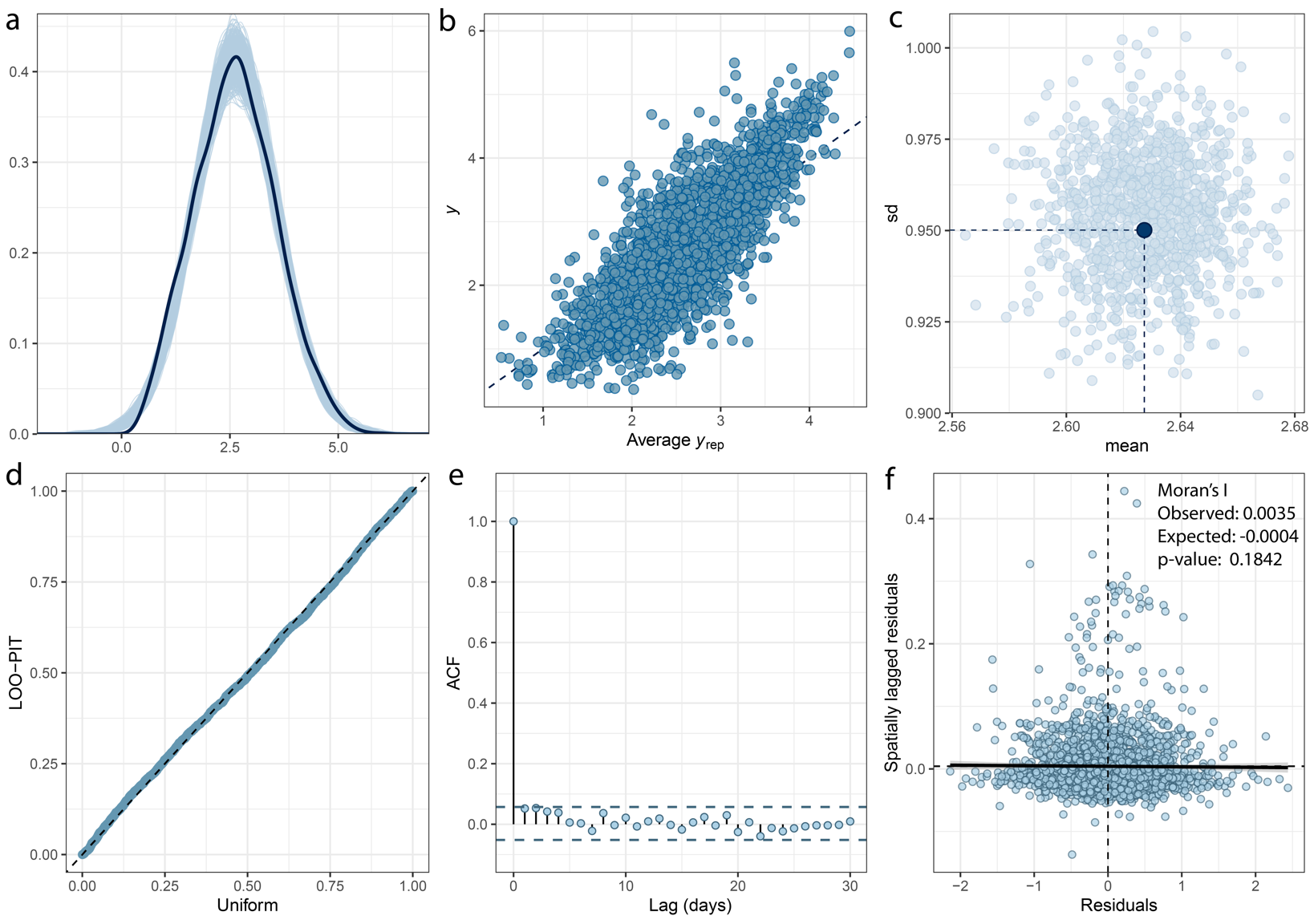
Supplementary Figure 3.** Posterior predictive checks for the Bayesian regression model explaining changes in daily distances travelled by wolves. (a) Kernel density estimate of the observed daily distances (black line) with kernel density estimates for 1000 simulated values (light blue lines) drawn from the posterior distribution; (b) observed daily distances vs predicted values; (c) mean and standard deviation of simulated daily distances; (d) calibration of marginal predictions using probability integral transformation (PIT) and approximated leave-one-out (LOO); (e) Temporal Autocorrelation Function (ACF) values for residuals of the Bayesian regression model, with dashed lines indicating the 95% confidence interval; and (f) spatial autocorrelation analysis and Moran scatterplot showing the relationship between residuals and spatially lagged residuals. Results of Moran's I test are included, with observed value, expected value, standard deviation, and significance (p-value).

**
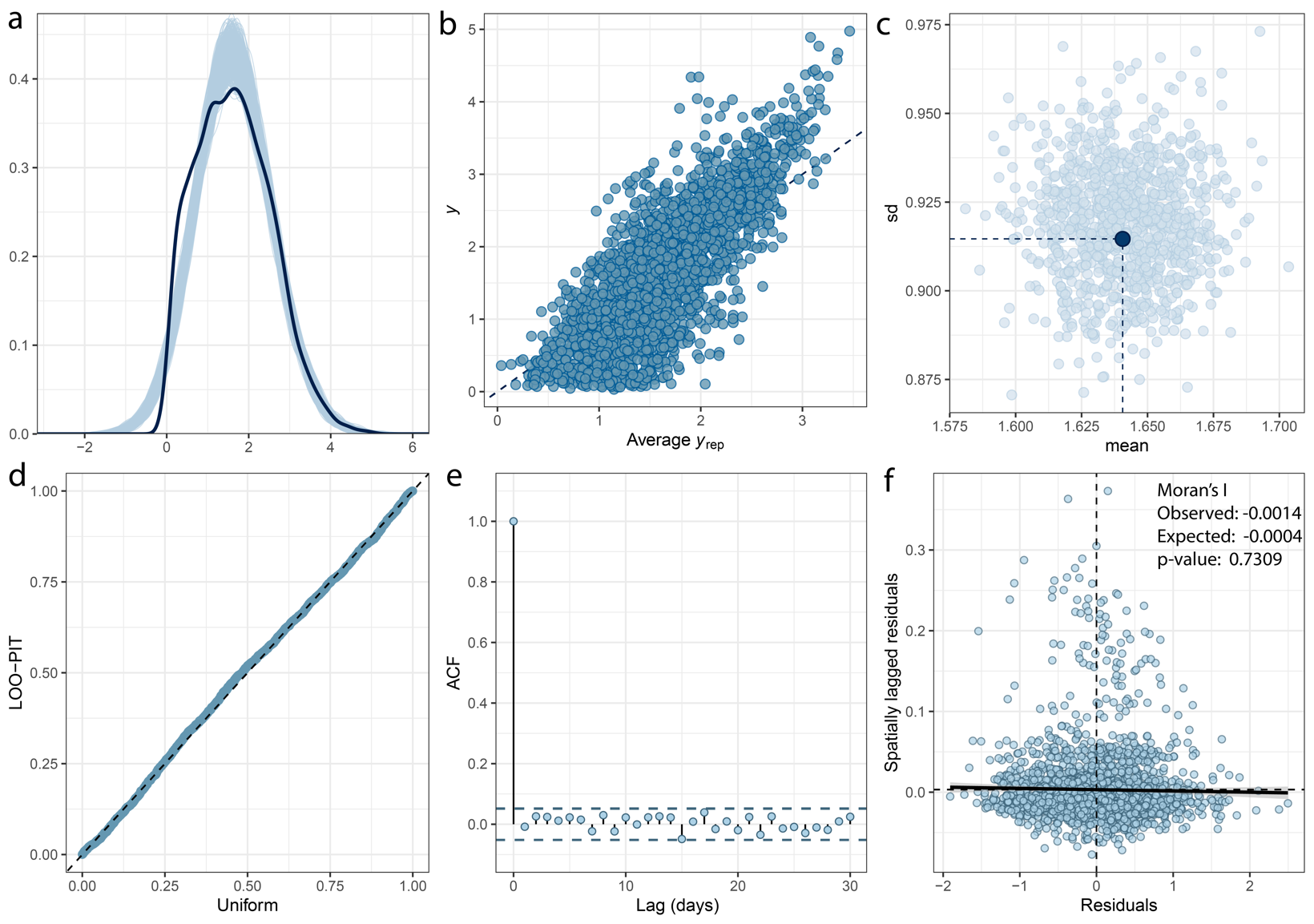
Supplementary Figure 4.** Posterior predictive checks for the Bayesian regression model explaining changes in net displacements. (a) Kernel density estimate of the observed net displacements (black line) with kernel density estimates for 1000 simulated values ((light blue lines)) drawn from the posterior distribution; (b) observed net displacements vs predicted values; (c) mean and standard deviation of simulated net displacements; (d) calibration of marginal predictions using probability integral transformation (PIT) and approximated leave-one-out (LOO); (e) Temporal Autocorrelation Function (ACF) values for residuals of the Bayesian regression model, with dashed lines indicating the 95% confidence interval; and (f) spatial autocorrelation analysis and Moran scatterplot showing the relationship between residuals and spatially lagged residuals. Results of Moran's I test are included, with observed value, expected value, standard deviation, and significance (p-value).

**
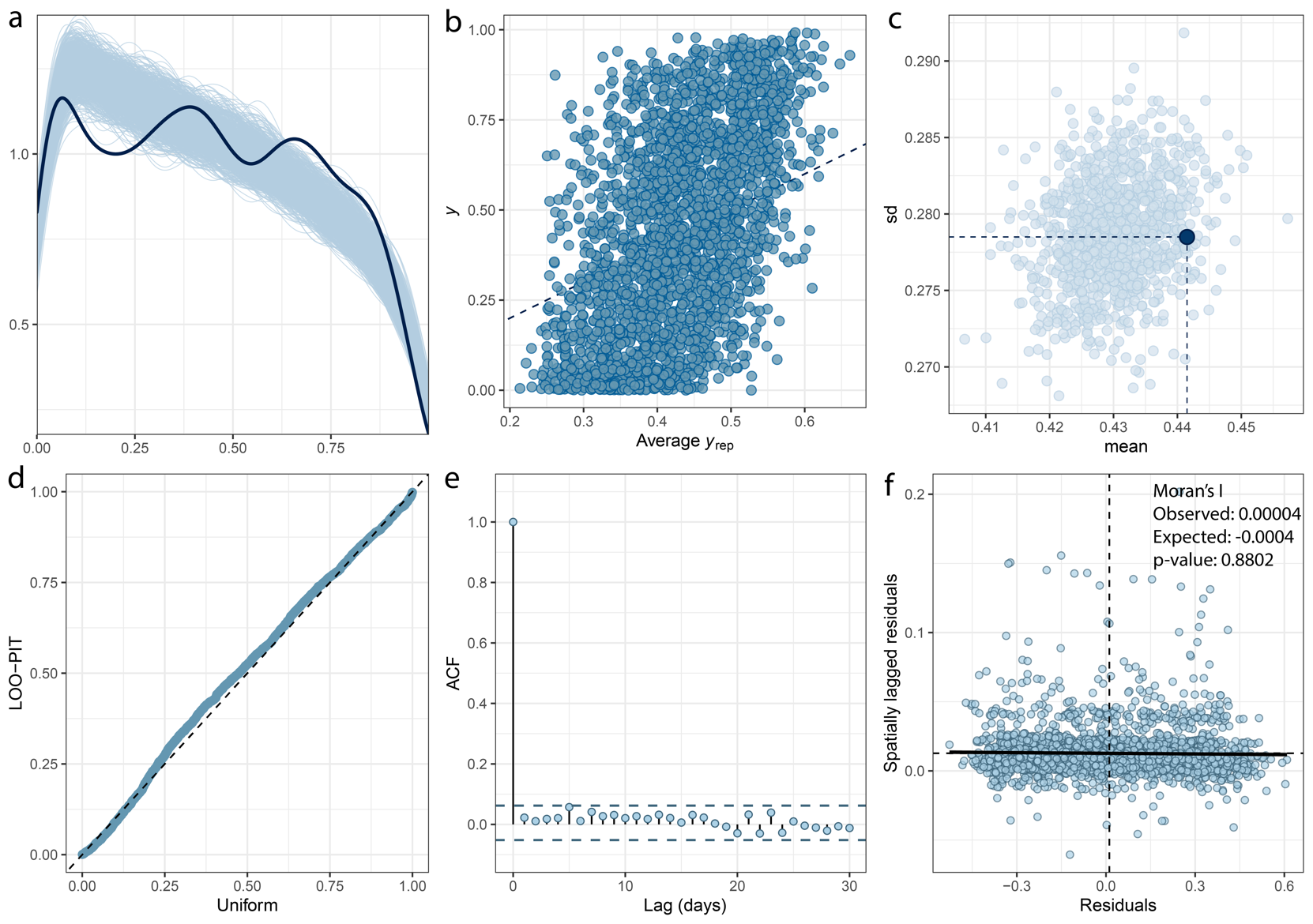
Supplementary Figure 5.** Posterior predictive checks for the Bayesian regression model explaining changes in net displacements. (a) Kernel density estimate of the observed net displacements (black line) with kernel density estimates for 1000 simulated values ((light blue lines)) drawn from the posterior distribution; (b) observed net displacements vs predicted values; (c) mean and standard deviation of simulated net displacements; (d) calibration of marginal predictions using probability integral transformation (PIT) and approximated leave-one-out (LOO); (e) Temporal Autocorrelation Function (ACF) values for residuals of the Bayesian regression model, with dashed lines indicating the 95% confidence interval; and (f) spatial autocorrelation analysis and Moran scatterplot showing the relationship between residuals and spatially lagged residuals. Results of Moran's I test are included, with observed value, expected value, standard deviation, and significance (p-value).
