# Supplementary material for: Human disturbance, preys and refuge cover shape top predator movements in anthropogenic landscapes
Source: Behav Ecol. 2026 Apr 13;37(3):arag037. doi: 10.1093/beheco/arag037 (PMC13121897; doi:10.1093/beheco/arag037)
Supplement: arag037_Supplementary_Data [file arag037_supplementary_data.zip › CLEAN_Additional_File_2_Supp_Tables_Wolf_Movement_Manuscript.docx]

**Supplementary Tables.**

**Supplementary Table 1.** Descriptive data of GPS sampling for each of the monitored wolves in this study to fit HMMs. Wolves L04, L21 and L43 were pack members until 15/10/2006, 01/01/2012 and 28/04/2014, respectively. From these dates, we considered them as non-pack members for analysis.

| ID | Age | Sex | Pack | Start Date | End Date | Total Days |
| --- | --- | --- | --- | --- | --- | --- |
|  |  |  |  |  |  |  |
| L01 | Adult | Male | Yes | 24/04/2006 | 25/09/2006 | 154 |
| L02 | Subadult | Male | No | 27/08/2006 | 18/10/2006 | 52 |
| L04 | Subadult | Male | Yes-No | 16/09/2006 | 20/12/2006 | 95 |
| L05 | Subadult | Female | Yes | 27/03/2007 | 03/12/2007 | 251 |
| L06 | Subadult | Male | Yes | 14/07/2007 | 17/10/2007 | 95 |
| L07 | Adult | Male | No | 18/10/2007 | 26/12/2007 | 69 |
| L08 | Subadult | Female | Yes | 16/07/2009 | 04/11/2009 | 111 |
| L09 | Adult | Male | Yes | 16/07/2009 | 25/06/2010 | 344 |
| L11 | Adult | Female | Yes | 29/07/2009 | 09/06/2010 | 315 |
| L12 | Subadult | Female | No | 14/08/2009 | 12/04/2010 | 241 |
| L13 | Subadult | Male | Yes | 19/11/2009 | 11/03/2010 | 112 |
| L16 | Subadult | Male | Yes | 22/10/2010 | 28/02/2011 | 129 |
| L17 | Subadult | Female | Yes | 11/04/2011 | 01/09/2011 | 143 |
| L18 | Subadult | Female | Yes | 11/04/2011 | 27/08/2011 | 138 |
| L19 | Subadult | Female | Yes | 05/04/2011 | 22/08/2011 | 139 |
| L20 | Adult | Female | Yes | 28/06/2011 | 27/10/2011 | 60 |
| L21 | Adult | Male | Yes-No | 10/07/2011 | 04/04/2012 | 269 |
| L22 | Subadult | Female | Yes | 14/07/2011 | 14/08/2012 | 397 |
| L23 | Subadult | Female | Yes | 13/08/2011 | 06/01/2012 | 146 |
| L24 | Adult | Female | Yes | 17/08/2011 | 16/11/2011 | 91 |
| L27 | Adult | Male | No | 27/12/2011 | 07/06/2012 | 178 |
| L28 | Adult | Male | Yes | 16/07/2012 | 02/04/2013 | 259 |
| L36 | Subadult | Male | Yes | 10/07/2013 | 13/05/2014 | 307 |
| L37 | Subadult | Male | Yes | 09/08/2013 | 16/06/2014 | 327 |
| L38 | Subadult | Male | Yes | 15/08/2013 | 24/01/2014 | 162 |
| L41 | Adult | Female | Yes | 12/03/2014 | 22/12/2014 | 285 |

**Supplementary Table 2.** Rationale for the inclusion and expected relationships of predictors in Bayesian Regression Models analyzing the factors influencing daily movements of Iberian wolves.

| Category | Predictor / Interaction | Rationale / Hypothesis |
| --- | --- | --- |
| **Intrinsic** | Age (Subadult vs. Adult) | We hypothesize that there will be no significant differences between subadult and adult wolves in terms of sinuosity, daily distances, and net displacement. Both subadult and adult wolves are expected to exhibit similar movement patterns overall. Nonetheless, we test the potential for age-related differences to emerge between these two age classes. |
| **Intrinsic** | Sex (Male vs. Female) | We hypothesize that there will be no significant differences between males and females in terms of sinuosity, net displacement, and daily distances. Both sexes are expected to exhibit similar movement patterns overall. Nonetheless, we test the potential for sex-related differences. |
| **Intrinsic** | Social Status (Pack vs. Non-pack member) | We expect that social status will significantly affect movement patterns, particularly in terms of sinuosity and net displacement. Pack members typically engage in more purposeful and constrained movements, as they frequently return to specific sites (rendezvous/den), which can limit net displacement and increase path directness. In contrast, non-pack members may exhibit more random, exploratory movement with longer net displacements and daily distances and higher sinuosity. |
| **Intrinsic** | Reproductive Period | We hypothesize that movement patterns will vary significantly across seasons (mating, reproductive, non-reproductive). We anticipate a reduction in daily distances and net displacement during the reproductive period due to denning and pup-rearing. |
| **Intrinsic** | **Sex * Reproductive Period** | We expect an interaction where females are likely to exhibit more pronounced reductions in movement (daily distance, net displacement and sinuosity) during the reproductive period compared to males due to their role in parturition and pup care. |
| **Intrinsic** | **Social Status * Reproductive Period** | We expect an interaction where pack members show reduced movement during the reproductive period due to cooperative care, while non-pack members do not. Furthermore, we expect non-pack members to demonstrate increased movement (net displacement, daily distances) during the mating period as a result of intensified search efforts for potential mates. |
| **Anthropogenic** | Human Population Density | We hypothesize a negative effect on wolf movement patterns. High human population density may lead to reduced daily distances and net displacement and higher sinuosity as wolves attempt to avoid human encounters. |
| **Anthropogenic** | Human Settlement Density | We hypothesize a negative effect; high settlement density could result in reduced daily distances and net displacement and higher sinuosity due to the increased likelihood of encountering humans. |
| **Anthropogenic** | **Population Density * Settlement Density** | We expect a compounding negative effect on wolf movement. In areas where both human population and settlement density are high, wolves are likely to reduce their daily distances and net displacements significantly. The interaction tests how the concentration versus dispersion of humans constrains movement. |
| **Anthropogenic** | Primary Road Density | We anticipate a pronounced negative effect on wolf movement (daily distance, net displacement) and increase in sinuosity due to high traffic volumes and the barrier effect. |
| **Anthropogenic** | Secondary Road Density | We anticipate a same effect on movement as primary roads, though potentially less severe than primary roads due to lower traffic and higher permeability. |
| **Anthropogenic/Refuge** | **Primary Road Density * Refuge PCA Dimension 1** | We anticipate an interaction where high refuge cover mitigates the negative impact of primary roads on the three facets of wolf movement. Ample refuge provides concealment, potentially allowing wolves to move more freely despite road presence. |
| **Anthropogenic/Refuge** | **Secondary Road Density * PCA Dimension 1** | We anticipate a similar buffering interaction for secondary roads, where cohesive and les fractal refuge cover can mitigate negative effects on daily distance, net displacement and straightness index. |
| Trophic resource | **Diet category** | We expect the primary prey type to significantly influence daily movement patterns. Wolves foraging on livestock are expected to exhibit shorter daily distances due to high prey density and aggregation. Wolves preying on wild ungulates or free-ranging horses are expected to travel longer distances due to more sparse and dispersed prey. |
| Landscape attribute | **Terrain Ruggedness Index** | We expect a negative relationship with both daily distances, net displacements. Rugged terrain increases the energy and effort required for movement, causing wolves to limit their travel. We further expect less sinuosity in movement trajectories as terrain ruggedness increases. |
| Refuge cover  (5 metrics synthesized into two PCA dimensions) | **PCA Dimension 1** | We hypothesize a positive effect on daily distances and net displacements, including also less tortuous movements. This dimension represents extensive, cohesive refuge, which offers enhanced shelter and potentially facilitates greater, more direct movement across the landscape. |
| Refuge cover  (5 metrics synthesized into two PCA dimensions) | **PCA Dimension 2** | This dimension represents fragmented refuge with many small, regularly shaped patches, which may hinder movement and lead to more interrupted, tortuous paths (affecting straightness, net displacement and daily distances). |
| Refuge cover  (5 metrics synthesized into two PCA dimensions) | **Refuge PCA Dimension 1 * Refuge PCA Dimension 2** | We included this interaction to test whether the benefits of extensive, cohesive refuge cover (PCA1) depend on the degree of fragmentation and shape complexity of that cover (PCA2). Specifically, we hypothesize that the positive effect of high PCA1 values (large, well-connected patches) on wolf movement (increased daily distance and net displacement) could be diminished or negated in landscapes where that same cover is also highly fragmented into many small, regularly shaped patches (high PCA2). |

**Supplementary Table 3.** Summary of the output from the Bayesian regression model explaining changes in daily distances travelled by wolves. Estimate = Median Posterior Value; SE = standard error; lower and upper CI = 95% credible interval values; PD = Probability of Direction; Rhat = R-hat convergence diagnostic; Bulk ESS = Bulk Effective Sample Size; Tail ESS = Tail Effective Sample Size. Significant fixed effects, defined as those with a 95% credible interval not crossing zero, are highlighted in bold.

| Term | Factor | Est. | SE | Lower 95% CI | Upper 95% CI | PD | Rhat | Bulk ESS | Tail ESS |
| --- | --- | --- | --- | --- | --- | --- | --- | --- | --- |
| Intercept | **None** | **3.02** | **0.2** | **2.63** | **3.41** | **1** | **1** | **1960.77** | **2725.29** |
| Age (Subadult) | Intrinsic | -0.05 | 0.18 | -0.39 | 0.31 | 0.62 | 1 | 1852.41 | 2430.19 |
| Sex (Male) | Intrinsic | -0.24 | 0.18 | -0.59 | 0.12 | 0.91 | 1 | 2064.32 | 2753.87 |
| Status (Non pack) | Intrinsic | -0.05 | 0.18 | -0.39 | 0.31 | 0.61 | 1 | 2527.81 | 3083.65 |
| Reproductive period (Non Reproductive) | Intrinsic | -0.02 | 0.06 | -0.14 | 0.1 | 0.62. | 1 | 3122.52 | 3496.42 |
| Reproductive period (Reproductive) | Intrinsic | -0.09 | 0.06 | -0.2 | 0.02 | 0.95 | 1 | 3088.59 | 3108.98 |
| Diet Category (Livestock) | **Trophic** | **-0.44** | **0.2** | **-0.82** | **-0.04** | **0.98** | **1** | **1709.33** | **2607.36** |
| Diet Category (Wild prey) | **Trophic** | 0.18 | 0.26 | -0.33 | 0.7 | 0.78 | 1 | 2404.09 | 2960.21 |
| Population density | **Anthropogenic** | **0.23** | **0.04** | **0.15** | **0.3** | **1** | **1** | **2426.59** | **3251.67** |
| Settlement density | **Anthropogenic** | **0.24** | **0.03** | **0.19** | **0.29** | **1** | **1** | **2940.03** | **3663.42** |
| Primary road density | **Anthropogenic** | **-0.08** | **0.02** | **-0.13** | **-0.04** | **1** | **1** | **3142.8** | **3278.4** |
| Secondary road density | **Anthropogenic** | **-0.14** | **0.02** | **-0.18** | **-0.11** | **1** | **1** | **3493.75** | **3281.8** |
| Refuge PCA Dim1 | **Refuge** | **0.07** | **0.02** | **0.04** | **0.11** | **1** | **1** | **2547.89** | **3238.38** |
| Refuge PCA Dim2 | **Refuge** | **0.14** | **0.02** | **0.1** | **0.18** | **1** | **1** | **3168.13** | **3362.6** |
| Terrain Ruggedness Index | **Landscape** | **-0.28** | **0.04** | **-0.35** | **-0.2** | **1** | **1** | **2561.84** | **3031.29** |
| Sex (Male) : Reproductive Period (Non Reproductive) | Intrinsic | -0.03 | 0.1 | -0.22 | 0.16 | 0.64 | 1 | 2952.87 | 3304.39 |
| Sex (Male) : Reproductive Period (Reproductive) | Intrinsic | 0.1 | 0.09 | -0.08 | 0.28 | 0.86 | 1 | 3164.92 | 3628.68 |
| Status (Non pack) : Reproductive Period (Non Reproductive) | **Intrinsic** | **0.47** | **0.15** | **0.18** | **0.75** | **1** | **1** | **2986.75** | **3561.21** |
| Status (Non pack): Reproductive Period (Reproductive) | **Intrinsic** | **0.43** | **0.15** | **0.13** | **0.73** | **1** | **1** | **2883.99** | **3325.74** |
| Population density : Settlement Density | **Anthropogenic** | **-0.1** | **0.01** | **-0.13** | **-0.07** | **1** | **1** | **2466.96** | **3252.38** |
| Primary road density : Refuge PCA Dim1 | **Anthropogenic** | **0.05** | **0.01** | **0.03** | **0.07** | **1** | **1** | **3399.23** | **3545.71** |
| Secondary road density : Refuge PCA Dim1 | **Anthropogenic/Refuge** | **0.07** | **0.01** | **0.05** | **0.09** | **1** | **1** | **3511.77** | **3701.97** |
| Refuge PCA Dim1 : Refuge PCA Dim2 | **Anthropogenic/Refuge** | **0.04** | **0.01** | **0.02** | **0.05** | **1** | **1** | **3242.67** | **3271.46** |

**Supplementary Table 4.** Summary of the output from the Bayesian regression model explaining changes in wolves' net displacements. Estimate = Median Posterior Value; SE = standard error; lower and upper CI = 95% credible interval values; PD = Probability of Direction; Rhat = R-hat convergence diagnostic; Bulk ESS = Bulk Effective Sample Size; Tail ESS = Tail Effective Sample Size. Significant fixed effects, defined as those with a 95% credible interval not crossing zero, are highlighted in bold.

| Term | Factor | Est. | SE | Lower 95% CI | Upper 95% CI | PD | Rhat | Bulk ESS | Tail ESS |
| --- | --- | --- | --- | --- | --- | --- | --- | --- | --- |
| Intercept | **None** | 1.83 | 0.16 | 1.51 | 2.13 | 1 | 1 | 2026.91 | 2806.92 |
| Age (Subadult) | Intrinsic | 0.03 | 0.14 | -0.24 | 0.31 | 0.58 | 1 | 2491.85 | 2960.98 |
| Sex (Male) | Intrinsic | -0.04 | 0.15 | -0.33 | 0.26 | 0.59 | 1 | 2477.89 | 2973.59 |
| Status (Non pack) | Intrinsic | -0.04 | 0.17 | -0.36 | 0.29 | 0.59 | 1 | 2912.9 | 3651.14 |
| Reproductive period (Non Reproductive) | Intrinsic | 0.08 | 0.06 | -0.04 | 0.2 | 0.91 | 1 | 3370.63 | 3646.14 |
| Reproductive period (Reproductive) | Intrinsic | -0.02 | 0.06 | -0.13 | 0.08 | 0.63 | 1 | 3349.87 | 3586.18 |
| Diet Category (Livestock) | **Trophic** | **-0.33** | **0.15** | **-0.64** | **-0.03** | **0.99** | **1** | **2216.41** | **3011.49** |
| Diet Category (Wild prey) | Trophic | 0.2 | 0.2 | -0.19 | 0.6 | 0.84 | 1 | 3138.52 | 3486.24 |
| Population density | **Anthropogenic** | **0.18** | **0.04** | **0.1** | **0.25** | **1** | **1** | **2917.94** | **3391.09** |
| Settlement density | **Anthropogenic** | **0.18** | **0.03** | **0.13** | **0.23** | **1** | **1** | **3403.02** | **3546.74** |
| Primary road density | Anthropogenic | -0.03 | 0.02 | -0.07 | 0.01 | 0.91 | 1 | 3653.44 | 3746.16 |
| Secondary road density | **Anthropogenic** | **-0.07** | **0.02** | **-0.11** | **-0.03** | **1** | **1** | **3172.51** | **3575.68** |
| Refuge PCA Dim1 | Refuge | 0.02 | 0.02 | -0.03 | 0.06 | 0.78 | 1 | 3211.67 | 3405.39 |
| Refuge PCA Dim2 | **Refuge** | **0.08** | **0.02** | **0.04** | **0.12** | **1** | **1** | **3232.78** | **3306.32** |
| Terrain Ruggedness Index | **Landscape** | **-0.15** | **0.04** | **-0.23** | **-0.08** | **1** | **1** | **3002.44** | **3509.68** |
| Sex (Male) : Reproductive Period (Non Reproductive) | Intrinsic | -0.11 | 0.1 | -0.29 | 0.09 | 0.86 | 1 | 3279.83 | 3318.1 |
| Sex (Male) : Reproductive Period (Reproductive) | Intrinsic | -0.04 | 0.09 | -0.23 | 0.15 | 0.66 | 1 | 3311.37 | 3701.83 |
| Status (Non pack) : Reproductive Period (Non Reproductive) | **Intrinsic** | **0.39** | **0.15** | **0.08** | **0.68** | **1** | **1** | **3165.31** | **3419.5** |
| Status (Non pack): Reproductive Period (Reproductive) | **Intrinsic** | **0.33** | **0.15** | **0.03** | **0.64** | **0.99** | **1** | **3059.24** | **3210** |
| Population density : Settlement Density | **Anthropogenic** | **-0.07** | **0.01** | **-0.1** | **-0.04** | **1** | **1** | **3023.38** | **3335.86** |
| Primary road density : Refuge PCA Dim1 | **Anthropogenic** | **0.04** | **0.01** | **0.02** | **0.07** | **1** | **1** | **3273.75** | **3465.99** |
| Secondary road density : Refuge PCA Dim1 | **Anthropogenic/Refuge** | **0.05** | **0.01** | **0.03** | **0.08** | **1** | **1** | **3446.97** | **3664.51** |
| Refuge PCA Dim1 : Refuge PCA Dim2 | **Anthropogenic/Refuge** | **0.02** | **0.01** | **0** | **0.03** | **0.97** | **1** | **3549.81** | **3724.25** |

| Term | Factor | Est. | SE | Lower 95% CI | Upper 95% CI | PD | Rhat | Bulk ESS | Tail ESS |
| --- | --- | --- | --- | --- | --- | --- | --- | --- | --- |
| Intercept | **None** | **-0.29** | **0.14** | **-0.57** | **-0.02** | **0.98** | **1** | **3165.59** | **3548.05** |
| Age (Subadult) | Intrinsic | 0.05 | 0.12 | -0.18 | 0.28 | 0.66 | 1 | 3295.57 | 3666.92 |
| Sex (Male) | Intrinsic | 0.01 | 0.11 | -0.21 | 0.24 | 0.55 | 1 | 3571.66 | 3455.68 |
| Status (Solitary) | Intrinsic | 0.09 | 0.2 | -0.29 | 0.47 | 0.68 | 1 | 3223.25 | 3550.79 |
| Reproductive period (Non reproductive) | Intrinsic | 0.08 | 0.07 | -0.07 | 0.22 | 0.86 | 1 | 3192.94 | 3334 |
| Reproductive period (Reproductive) | Intrinsic | 0 | 0.07 | -0.14 | 0.13 | 0.52 | 1 | 3406.81 | 3177.23 |
| Diet category (Livestock) | Trophic | -0.15 | 0.13 | -0.4 | 0.1 | 0.88 | 1 | 3261.09 | 3394.79 |
| Diet category (Wild prey) | Trophic | 0.02 | 0.17 | -0.32 | 0.37 | 0.54 | 1 | 3232.85 | 3390.44 |
| Population density | Anthropogenic | 0.04 | 0.05 | -0.05 | 0.13 | 0.81 | 1 | 3595.11 | 3408.22 |
| Settlement density | Anthropogenic | 0.06 | 0.04 | -0.01 | 0.13 | 0.96 | 1 | 3313.53 | 3774.54 |
| Primary road density | Anthropogenic | 0 | 0.03 | -0.05 | 0.06 | 0.5 | 1 | 3479.94 | 3461.62 |
| Secondary road density | Anthropogenic | 0.01 | 0.03 | -0.04 | 0.06 | 0.66 | 1 | 3408.54 | 3277.74 |
| Refuge PCA Dim1 | Refuge | -0.03 | 0.02 | -0.08 | 0.02 | 0.91 | 1 | 3472.78 | 3387.28 |
| Refuge PCA Dim2 | Refuge | 0.04 | 0.03 | -0.02 | 0.09 | 0.9 | 1 | 3134.54 | 3178.43 |
| Terrain Ruggedness Index | Landscape | -0.03 | 0.04 | -0.12 | 0.05 | 0.77 | 1 | 3728.92 | 3418.32 |
| Status (Non pack) : Reproductive Period (Non Reproductive) | Intrinsic | 0.14 | 0.2 | -0.25 | 0.53 | 0.77 | 1 | 3456.52 | 3507.28 |
| Status (Non pack): Reproductive Period (Reproductive) | Intrinsic | 0.12 | 0.19 | -0.26 | 0.49 | 0.72 | 1 | 3540.92 | 3232.42 |
| Population density : Settlement Density | Intrinsic | -0.01 | 0.02 | -0.04 | 0.03 | 0.64 | 1 | 3368.05 | 3772.8 |
| Primary road density : Refuge PCA Dim1 | **Intrinsic** | **0.03** | **0.01** | **0** | **0.05** | **0.98** | **1** | **3383.89** | **3235.2** |
| Secondary road density : Refuge PCA Dim1 | Anthropogenic/Refuge | 0.02 | 0.01 | -0.01 | 0.05 | 0.91 | 1 | 3443.44 | 3563.09 |
| Refuge PCA Dim1 : Refuge PCA Dim2 | Anthropogenic/Refuge | 0.01 | 0.01 | -0.02 | 0.03 | 0.71 | 1 | 3434.41 | 3455.21 |

**Supplementary Table 5.** Summary of the output from the Bayesian regression model explaining changes in wolves' net displacements. Estimate = Median Posterior Value; SE = standard error; lower and upper CI = 95% credible interval values; PD = Probability of Direction; Rhat = R-hat convergence diagnostic; Bulk ESS = Bulk Effective Sample Size; Tail ESS = Tail Effective Sample Size. Significant fixed effects, defined as those with a 95% credible interval not crossing zero, are highlighted in bold.
